# NO homeostasis is a key regulator of early nitrate perception and root elongation in maize*

**DOI:** 10.1093/jxb/ert358

**Published:** 2013-11-12

**Authors:** Alessandro Manoli, Maura Begheldo, Andrea Genre, Luisa Lanfranco, Sara Trevisan, Silvia Quaggiotti

**Affiliations:** ^1^Department of Agriculture, Food, Natural Resources, Animals and Environment (DAFNAE), University of Padua, Agripolis, Viale dell’Università, 16, 35020 Legnaro (PD), Italy; ^2^Department of Life Sciences and Systems Biology, University of Turin, Viale Mattioli 25, 10125 Turin, Italy

**Keywords:** Maize, nitrate, nitrate reductase, nitric oxide, non-symbiotic haemoglobin, root, transition zone.

## Abstract

Crop plant development is strongly dependent on nitrogen availability in the soil and on the efficiency of its recruitment by roots. For this reason, the understanding of the molecular events underlying root adaptation to nitrogen fluctuations is a primary goal to develop biotechnological tools for sustainable agriculture. However, knowledge about molecular responses to nitrogen availability is derived mainly from the study of model species. Nitric oxide (NO) has been recently proposed to be implicated in plant responses to environmental stresses, but its exact role in the response of plants to nutritional stress is still under evaluation. In this work, the role of NO production by maize roots after nitrate perception was investigated by focusing on the regulation of transcription of genes involved in NO homeostasis and by measuring NO production in roots. Moreover, its involvement in the root growth response to nitrate was also investigated. The results provide evidence that NO is produced by nitrate reductase as an early response to nitrate supply and that the coordinated induction of non-symbiotic haemoglobins (nsHbs) could finely regulate the NO steady state. This mechanism seems to be implicated on the modulation of the root elongation in response to nitrate perception. Moreover, an improved agar-plate system for growing maize seedlings was developed. This system, which allows localized treatments to be performed on specific root portions, gave the opportunity to discern between localized and systemic effects of nitrate supply to roots.

## Abstract (precis)

Nitrate reductase produces nitric oxide (NO) as an early response to nitrate, and the coordinated induction of ns-haemoglobins finely modulates NO level. The control of NO homeostasis regulates root elongation and represents a novel key component of nitrate signaling in maize

## Introduction

Soil nutrient acquisition intensely affects global crop production (Forde and Clarkson, 1999; Robertson and Vitousek, 2009). In poor nations drought and low soil fertility cause low yields and food insecurity, while in rich nations intensive fertilization leads to leaching of nutrients and/or greenhouse gas emission (Donner and Kucharik, 2008). The development of new crop cultivars with enhanced soil resource acquisition is therefore an important strategic goal for modern agriculture (Lynch, 1998, 2007; Vance *et al.*, 2003). Understanding nutrient responses at the organism level will be useful to modify plant metabolism, physiology, growth, and developmental programmes to improve nutrient use efficiency and productivity in crops.

The macronutrient nitrogen is essential for plant growth and development as it is a component of proteins, nucleic acids, and many cofactors and secondary metabolites. In aerobic soils, nitrate is the major source of nitrogen for most plant species (Ahmad *et al.*, 2007; Nischal *et al.*, 2012).

Plants have the potential for adaptation to dramatic fluctuations of nitrogen availability by modulating their capacity for nutrient acquisition and by altering whole-plant morphology and metabolism, such as increasing the root/shoot ratio (Rubio *et al.*, 2009). Developmental adaptive mechanisms stimulate growth in organs that directly participate in nutrient acquisition, such as primary roots (Walch-Liu and Forde, 2008). A dual effect of external nitrate on root system architecture development has been depicted in the model species *Arabidopsis thaliana*: (i) a systemic inhibition of lateral primordia by uniformly high nitrate concentrations at a post-emergence stage; and (ii) a localized stimulation of elongation in N-starved roots at the site of contact with a nitrate-rich supply, known as the foraging capacity (Zhang and Forde, 1998; Zhang *et al.*, 1999, 2007; Linkohr *et al.*, 2002; Ruffel *et al.*, 2011). Apart from a few known pathways that involve transcription factors, micro-RNAs, hormonal signals, and, more recently, nitrate transporters with dual affinity for nitrate and auxin (Little *et al.*, 2005; Remans *et al.*, 2006; Chiou, 2007; Miller *et al.*, 2007; Gifford *et al.*, 2008; Krouk *et al.*, 2010, 2011; Vidal *et al.*, 2010; Castaings *et al.*, 2011; Rubio-Somoza and Weigel, 2011; Ruffel *et al.*, 2011; Xu *et al.*, 2011; Trevisan *et al.*, 2012), the understanding of the sensing of external nitrate conditions and of the signal transduction system that leads to an altered development of roots is still poor.

To trigger adaptive responses and to induce fast switching from starvation metabolism to nutrient assimilation, nitrate itself or its primary assimilation products serve as signalling molecules (Schulze *et al.*, 1994; Crawford, 1995; Scheible *et al.*, 1997; Stitt, 1999; Gojon *et al.*, 2010). Significant advances have been made recently concerning the molecular mechanisms of NO_3_^–^ sensing and signalling in *Arabidopsis*, and the striking action of NO_3_^–^ as a signal in regulating genome expression has been unravelled (Bouguyon *et al.*, 2012). Prolonged nitrate starvation has been demonstrated to largely affect gene transcription, which produces effects on the early nitrate signalling mechanisms. Transcriptomic analyses have evidenced coregulated transcriptional patterns in maize root epidermal cells for genes putatively involved in nitric oxide (NO) synthesis/scavenging (Trevisan *et al.*, 2012).

Nitric oxide is a free radical that is considered to be a general plant signal, since it regulates both normal developmental processes and biotic or abiotic stress responses involving cross-talk with phytohormones (for reviews, see Durner and Klessig, 1999; Wojtaszek, 2000; Beligni and Lamattina, 2001; Lamattina *et al.*, 2003). NO has been reported to be required for root organogenesis (Pagnussat *et al.*, 2002), formation of adventitious roots (Pagnussat *et al.*, 2003), lateral root development (Correa-Aragunde *et al.*, 2004), and root hair formation (Lombardo *et al.*, 2006). Recently, Correa-Aragunde *et al.* (2004) suggested the possibility that auxin and NO might be on a linear signalling pathway in the process of lateral root formation in tomato. However, knowledge of the molecular mechanisms by which NO regulates growth and development is still fragmentary.

NO is produced in plant tissues by two major pathways, one enzymic and the other non-enzymic (Wendehenne *et al.*, 2004). The NO-producing enzymes identified in plants are nitrate reductase (NR) and several NO synthase-like proteins, including one localized in peroxisomes which has been biochemically characterized (del Río *et al*., 2004). Interestingly, it was recently shown that non-symbiotic haemoglobin nsHb 1 could reduce NO_2_^–^ to NO at a constant rate that was far in excess of that reported for other haemoglobins (Sturms *et al.*, 2011). Plant Hbs are able to regulate several NO effects, as recently reviewed by Hill (2012). Class II nsHbs contribute to NO removal when overexpressed (Hebelstrup *et al.*, 2006, 2012). Moreover, several studies have demonstrated a role for plant Hbs in catalysing the turnover of NO to nitrate (Dordas *et al.*, 2003*a*,*b*, 2004; Perazzolli *et al.*, 2004; Hebelstrup *et al.*, 2006, 2012).

The nitrate-regulated expression and the spatial distribution of NR and Hb transcripts in epidermal cells that have been recently evidenced in maize roots strongly suggest that they could play an important role during the early perception and signalling of nitrate in the rhizosphere (Trevisan *et al.*, 2011). Moreover the colocalization of mRNAs for NR and Hb observed in the root apex matches with the major sites of NO accumulation, as shown in *Arabidopsis* (Stöhr and Stremlau, 2006), suggesting that these two genes may represent the pivotal elements of a finely tuned system for NO homeostasis and signalling.

The involvement of NO in the nitrate signalling pathway opens a wide field of research. This report evaluated the contribution of NO in the nitrate-regulated pathway that directs root system architecture, unravelling the role of NO as a nitrate-related signal. The study focused on both the characterization of the expression profiles of selected genes putatively involved in NO homeostasis and the determination of NO production by roots in response to different N treatments. In addition, since the genes therein selected have proved to be very good candidates for monitoring nitrate sensing in maize roots, they are proposed to be early physiological molecular markers for the response to this anion. Furthermore, this study also deepened the knowledge of the effect of nitrate on root growth and especially on root elongation. Finally, an improved agar-plate culture system for studying the *Zea mays* L. root response to nutrients has been developed. Thanks to this system, it has been possible to discriminate between localized and systemic effects of nitrate supply to roots. Overall, the data provide evidence that, in maize roots, NO is produced by NR as an early response to nitrate supply. Moreover, the coordinated induction of nsHbs finely regulate its steady-state level. The control of the NO production by the synergic action of NR and nsHbs would seem, moreover, to participate to the complex signalling network involved in the modulation of root growth in response to nitrate.

## Materials and methods

### Maize growth and experimental design

**Fig. 1.**
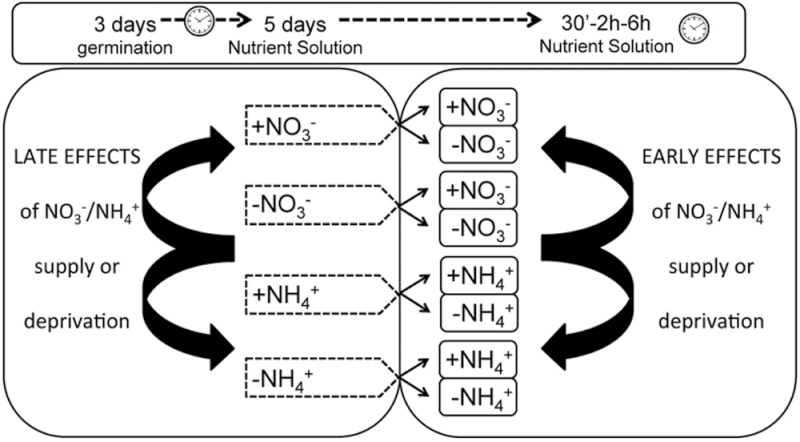
Workflow model of the experimental conditions. Seeds were sowed on filter paper, and 3 days after germination seedlings were divided into four groups and transferred for 5 days to four different hydroponic solutions: +N solutions (+NO_3_^–^ and +NH_4_^+^ and –N solutions (NO_3_^–^ and NH_4_^+^-depleted nutrient solution). After 5 days, seedlings were transferred to different nutrient solutions (+N solutions and –N solutions) and were treated for different times (30min, 2h, and 6h). At the end of the treatments, the eight groups were used to compare the effects of long- and short-term of nitrogen supply/depletion by means of a multifaceted transcriptomic approach.

Seeds of maize inbred line B73 were sown and then transferred to nutrient solution as described by Quaggiotti *et al.* (2003). For a first set of expression analyses, seedlings were grown in different nutrient solutions for 5 days and then treated for a few hours, as described in Fig. 1. Nitrate, ammonium, and ammonium nitrate were supplied at a concentration of 1mM. In the nitrogen-depleted nutrient solution, KNO_3_ was replaced by 1mM KCl and NH_4_SO_4_ by MgSO_4_.

For NO content measurement, for subsequent expression analyses, and for the analysis of root elongation rate, seedlings were grown for 24h in the nutrient solution to allow the manipulation of younger roots. To deepen the understanding of the role of NO in maize root response to nitrate, 1mM sodium tungstate dihydrate (Na_2_WO_4_.2H_2_O), 1mM 2-(4-carboxyphenyl)- 4,4,5,5-tetramethylimidazoline-1-oxyl-3-oxide (cPTIO), 0.2mM l-NG-nitroarginine methyl ester (l-NAME), 0.01mM sodium nitroprusside (SNP), or 1mM (±)-(E)-4-ethyl-2-[(E)-hydroxyimino]-5-nitro-3-hexenamide (NOR) were supplied to the nutrient solution (either NO_3_^–^-supplied or NO_3_^–^-deprived).

Seedlings of the same age were also utilized to evaluate the expression of selected genes in four different portions of roots, as indicated by Baluška *et al*. (2010), after nitrate supply. The four zones sampled were the root meristem (4mm from the root tip), the transition zone (the next 1cm), the elongation zone (the next 1cm), and the maturation zone (the residual portion). Roots were harvested after 2h of nitrate provision and the four fragments were immediately cut and frozen, both for the treatment and the negative control (–NO_3_^–^).

### Growth of maize seedlings in agar medium

**Fig. 2.**
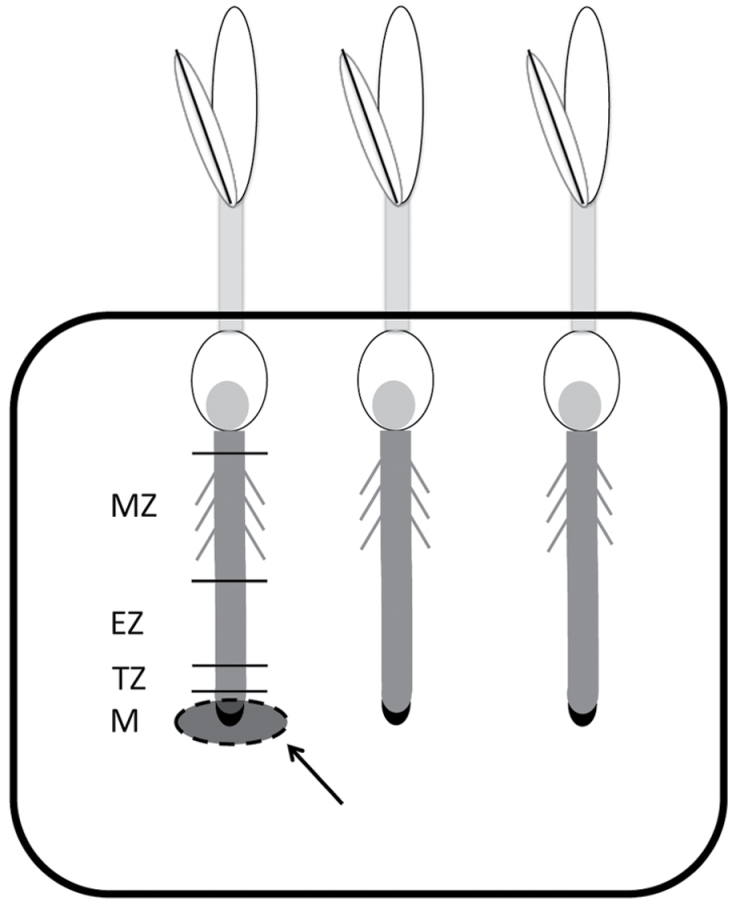
Design of the split-root system used to investigate the localized effects of nitrate on the intact root apex of maize seedlings. Seeds of maize inbred line B73 were sown on paper and then seedlings were transferred to a vertical plate system. Plate prepared with N-depleted solution and 1% agar were either supplied with 1mM nitrate (+N) or depleted (–N) by cutting and replacing a rounded portion of the agar, thus only apical portion of the root system could perceive the change of treatment. Seedlings continued to grow after the replacement of the rounded portion of agar, and at the end of the treatment they were removed from the system and harvested. M, meristem; TZ, transition zone; EZ, elongation zone; MZ, maturation zone.

An improved method was developed to grow maize seedlings on agar. To this aim, plastic boxes (17.9×12.9×2.6cm) modified with suitable holes on one side were utilized (Supplementary Fig. S1, available at *JXB* online). This system permitted the insertion of young roots, which can grown vertically along the agar medium allowing the shoot to develop outside the box and enabled us to perform localized treatments on single root portions, as described in Fig. 2. The agar concentration utilized was 1% after a preliminary test with concentrations ranging from 0.8 to 1.2%. The nutrients were supplied as indicated for hydroponics.

Roots of seedlings grown for 24h in a nitrate-depleted agar plate were transferred on an identical medium to which, in correspondence to specific root regions, round slices (about 1–1.5cm in diameter) of agar were removed and substituted with new ones containing 1mM nitrate. For the negative control, the slices were substituted with new nitrate-depleted ones to subject roots to a similar mechanical stress, thus avoiding false positives due to the perception of the discontinuance of agar and not to the nitrate presence.

### Morphological analyses

For the analysis with WinRhizo, germinated seeds of maize inbred line B73 were transferred to 2-l tanks containing five different aerated nutrient solutions (changed every 2 days) according to the treatments—(a) +NO_3_^–^, (b) –NO_3_^–^, (c) +NH_4_^+^, (d) –NH_4_^+^, and (e) +NH_4_NO_3_—and were then placed in a growth chamber for 8 days. Morphological analyses, including total root length (cm), total surface area (cm^2^), mean root diameter (mm), and number of root tips, were performed on 30 randomly chosen plants for each treatment (two biological replicates) by means of a STD-1600 EPSON scanner set and an image analysis software (WinRhizo Pro, Regent Instruments, QC, Canada). Statistical analyses were performed using R version 2.14.2.

For the analysis of primary root elongation rate, seedlings were grown for 24h in a 500-ml beaker and subjected to six different treatments according to the growing medium, as follows: (a) +NO_3_^–^, (b) +NO_3_^–^ +cPTIO, (c) +NO_3_^–^ +tungstate, (d) –NO_3_^–^, (e) –NO_3_^–^ +SNP, (f) +NH_4_^+^. The measures of primary root length were made with a ruler on 16 seedlings for each group, in four independent biological repetitions. To investigate the possible effects of toxicity due to the use of chemicals, both total root weight and leaf weight were also measured. Statistical analyses were performed by using R version 2.14.2.

### RNA extraction and cDNA synthesis

Tissues used for gene expression analyses were collected and immediately frozen in liquid nitrogen and kept at −80 °C for subsequent RNA extraction.

Total RNA was extracted from 250mg frozen tissue as described by Trevisan *et al.* (2011) and using the TRIzol method (Invitrogen, San Giuliano Milanese, Italy). An aliquot of total RNA was treated with RQ1 RNAse-free DNAse (Promega, Milano, Italy) as described by Falchi *et al.* (2010). Total RNA (1 μl) was quantified using a Nanodrop 1000 (Thermo Scientific, Nanodrop Products, Wilmington, DE, USA). cDNA was synthesized from 500ng of total RNA mixed with 1 μl of 10 μM oligo-dT, as described by Manoli *et al.* (2012).

### Selection of genes to be evaluated, maize sequences identification, and primer design

The list of genes analysed is reported in Supplementary Table S1, together with the primers utilized for real-time quantitative PCR (RT-qPCR) expression analysis. They were chosen according to previously published results (Trevisan *et al.*, 2011, 2012). *Hb* (NCBI accession AF236080.1) and *NR1* (AF153448.1) were then chosen for further more detailed analysis and the analysis was extended to the expression of *Hb2* (NM_001112349.1), *NiR* (ACG29734.1), and *NOA1* (NM_001174573), which were selected by screening the B73 genome database (http://www.maizesequence.org/index.html), and *NRT2.1* (AY129953.1, Quaggiotti *et al.*, 2003), which was used as a positive control for nitrate perception. The *NOA1* sequence was identified based on its similarity with *AtNOA1* (At3g47450.1).

Primers were designed with Primer3 web tool (version 0.4.0; http://frodo.wi.mit.edu/primer3/; Rozen and Skaletsky, 2000) and further verified with the PRATO web tool (Nonis *et al.*, 2011; http://prato.daapv.unipd.it).

### Real-time quantitative PCR

Relative quantification of transcripts by RT-qPCR was performed in a StepOne Real-Time PCR System (Applied Biosystems, Monza, Italy) as described by Nonis *et al.* (2007). Experiments were conducted using SYBR Green chemistry (Applied Biosystems), according to the manufacturer’s instructions. For each reaction, 2.5ng retrotranscribed RNA was used as template. Three technical replicates were performed on six independent biological replicates using the conditions described by Trevisan *et al.* (2011). Melting curve analysis was performed to confirm the absence of multiple products and primer dimers. Data were exported and analysed according to the Livak and Schmittgen (2001) method using as reference genes *LUG* (leunig primers, forward 5′-TCCAGTGCTACAGGGAAGGT-3′ and reverse 5′- GTTAGTTCTTGAGCCCACGC-3′) and *MEP* (membrane protein PB1A10.07c, primers: forward 5′-TGTACTCGGCAATG CTCTTG-3′ and reverse 5′- TTTGATGCTCCAGGCTTACC-3′), according to Manoli *et al.* (2012). For each transcript, the ratio between the expression measured for a given treatment and of its own control was used to estimate up- or downregulation of genes. The ratios obtained were then expressed as base-2 logarithm to build the graphs.

### NO detection

Germinated seeds were transferred to a nitrogen-depleted nutrient solution, and after 24h root apices about 2cm long were excised and incubated for 30min in 2ml detection buffer (10mM Tris-HCl, pH 7.4) with 15 μM of 4,5-diaminofluorescein diacetate (DAF-2DA). Subsequently, the apices were washed twice for 5min with fresh detection buffer and placed on a microscope slide fixed with a Secure-Seal hybridization chamber gasket (Life Technologies, Carlsbad, CA, USA) (20-mm diameter, 0.8-mm deep) and analysed for NO production by stereo- and confocal microscopy. For each chamber, one apex was incubated (as will be described in detail).

For stereomicroscopy, the chambers were immediately filled with nutrient solution containing 1mM KNO_3_ (+NO_3_^–^) or nitrogen-depleted nutrient solution containing 1mM KCl (negative control, –NO_3_^–^) and examined by epifluorescence with a SteReo Lumar V.12 (Carl Zeiss, Oberkochen, Germany). Images were captured with an MRc5 Axiocam Zeiss colour camera every 5min for 50min and processed with Adobe Photoshop CS4 (Adobe, San Jose, CA, USA).

Confocal NO measurements were carried out filling the chambers alternatively with: (a) +NO_3_^–^ solution; (b) –NO_3_^–^solution; (c) +NO_3_^–^ nutrient solution supplied with the NO scavenger cPTIO; (d) –NO_3_^–^ nutrient solution supplemented with the NO donor NOR-3; (e) +NO_3_^–^ solution with sodium tungstate. The incubation with DAF-2DA was carried out as previously described.

All apices were observed with a Leica TCS-SP2 confocal microscope (Leica Microsystems CMS, Mannheim, Germany) and images were acquired every 5min for 45min from the beginning of the incubation. Images were then analysed using Leica confocal software. Normalization of the data and ratios of mean fluorescence intensities were calculated as described by Calcagno *et al.* (2012). Five root pieces were tested for each condition and five independent repeats were analysed for each treatment.

## Results

### Nitrate exerts specific effects on genes involved in NO homeostatic control

**Fig. 3.**
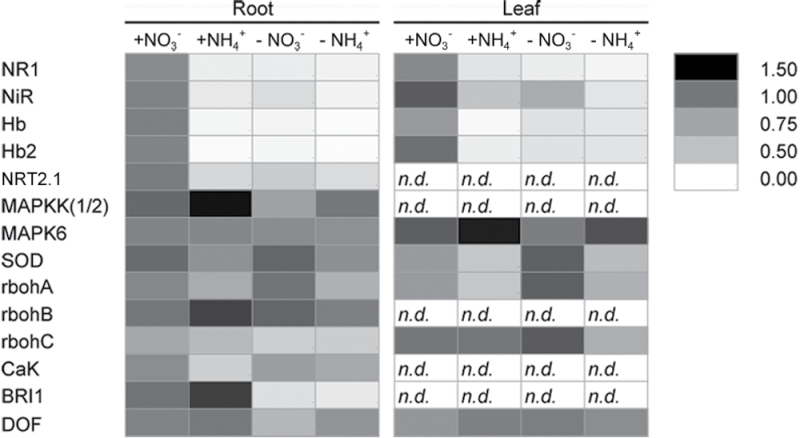
Heat map showing gene expression of 14 genes significantly regulated by long-term nitrate or ammonium supply and depletion in *Zea mays* L. roots and leaves. Seedlings were grown for 5 days in a nutrient solution containing 1mM nitrate (+NO_3_^–^) or 1mM ammonium (+NH_4_^+^) or N-deprived (–NO_3_^–^ and –NH_4_^+^). At the end of the treatment, seedlings were harvested and roots were separated from leaves. The grey scale represents the level of gene expression. Values are reported as arbitrary units and are the means of three technical repetitions performed on six independent biological replicates.

The expression of a number of previously identified genes (Quaggiotti *et al.*, 2003; Trevisan *et al.*, 2011, 2012), together with that of some new ones (Supplementary Table S1), was measured in roots and leaves of seedlings grown for 5 days in a nutrient solution containing 1mM nitrate (+NO_3_^–^), 1mM ammonium (+NH_4_^+^), or N-deprived (both –NO_3_^–^ and –NH_4_^+^) (Fig. 3).

The transcriptional response of five genes (*NR1*, *Hb*, *Hb2*, *NRT2.1*, *NiR*) evidenced a very strong nitrate responsiveness in roots. A similar behaviour was observed in leaves, even if to a lower extent. The rest of genes selected, on the contrary, did not evidence a specific nitrate responsiveness.

**Fig. 4.**
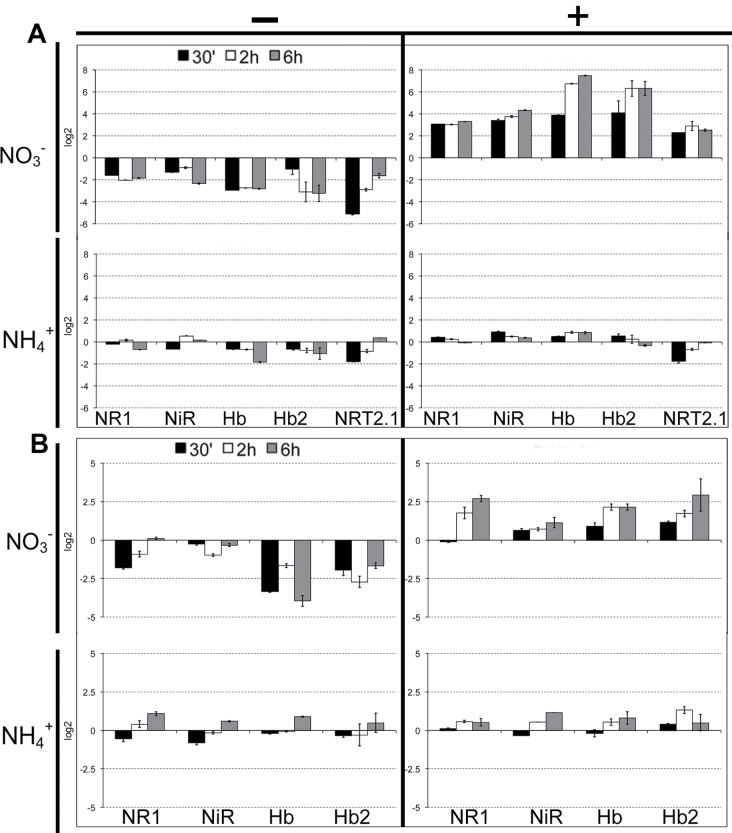
Time course of the expression of five genes significantly regulated by short-term nitrate/ammonium treatments in *Zea mays* L. roots (A) and leaves (B). Data are reported as base-2 logarithm of the ratio between the expression levels measured for samples subjected to the treatments, as described in Fig. 1 (short-term nitrogen starvation in seedlings grown in +N conditions and short-term nitrogen provision in seedlings grown in –N conditions) and of its own control. The left column (–) shows the differences in gene expression in seedlings supplied for 5 days with NO_3_^–^ (upper part) or NH_4_^+^ (lower part) and then deprived for 30min, 2h, and 6h; the right column (+) shows the differences of expression measured after resupply with NO_3_^–^ (upper panels) or NH_4_^+^ (lower panels) for 30min, 2h, and 6h in seedlings grown 5 days in a N-deprived medium.

The expression of the same set of genes was also assessed on root and leaf tissues of 5-day-old seedlings, but after only 30min, 2h, and 6h of nitrate/ammonium provision or depletion. The time course of the expression of the five nitrate-specific targets in both roots and leaves after a few hours of nitrate/ammonium supply/starvation is shown in Fig. 4 (the expression patterns of the other genes tested is reported in Supplementary Fig. S2).

The nitrate supply induced a significant increase of transcript accumulation for all five genes in both roots and leaves (Fig. 4A and 4B, upper panels), even if in roots it was much more noticeable (from 4–16-fold after 30min of NO_3_^–^ supply to 8–>100-fold after 6h). Conversely, the transcription of the five genes did not show a similar increase when ammonium was supplied as the form of nitrogen, neither in roots nor in leaves (Fig. 4A, B, lower panels), confirming the specificity of responsiveness to nitrate. Also in the case of N-deprivation, all five genes displayed a more evident response (decrease of expression) to nitrate deprivation in comparison to that measured for ammonium removal (Fig. 4A, B, left panels), both in leaves and in roots. These five specifically nitrate-inducible genes were thus selected for the subsequent and more detailed expression analyses.

### Root growth responds specifically to nitrate availability

The effect of nitrate supply on root development was evaluated in comparison to that of both ammonium and NO_3_NH_4_ in plants grown in nutrient solution for 5 days (Table 1 and Supplementary Fig. S4). The analysis of root length, root surface area, and number of tips evidenced a similar pattern, showing the strongest root growth stimulation in seedlings grown with 1mM nitrate. Values measured for these three parameters in plants grown with ammonium were significantly lower (50–60%) than those observed for nitrate-supplied roots and were closest to rates observed for NO_3_^–^-depleted roots (nitrate-negative control). Furthermore, an inhibitory effect of ammonium supply was visible for both root length and tip number, which showed values even lower with respect to the negative control. The supply of NO_3_NH_4_ slightly stimulated these three parameters, even if to a significantly lower extent with respect to nitrate.

**Table 1.** Effects of nitrate supply on root development after 5 days

Treatment	Length (cm)	Surface area (cm^2^)	Diameter (mm)	Tips (*n*)
NO_3_^–^ supply	79.48±3.98^*a*^	12.58±0.55^*a*^	0.50±0.01^*d*^	91.20±5.07^*a*^
NO_3_^–^ depletion	51.61±2.96^*c*^	8.60±0.46^*c*^	0.55±0.01^*c*^	67.40±4.57^*b*^
NH_4_ supply	36.59±1.39^*d*^	8.18±0.28^*c*^	0.70±0.01^*a*^	55.43±3.34^*c*^
NH_4_ depletion	51.34±2.15^*c*^	9.03±0.43^*c*^	0.52±0.01^*d*^	72.90±2.75^*b*^
NO_3_NH_4_ supply	60.08±3.56^*b*^	10.42±0.55^*b*^	0.61±0.01^*b*^	64.23±2.79^*b,c*^

Different letters indicate statistically significant differences among samples (*P* < 0.05, ANOVA).

The mean root diameter showed an opposite trend with the maximum rate observed for ammonium-treated roots and the lowest one for nitrate-supplied plants, which evidenced values even lower than those observed for nitrate-depleted roots. These observations, besides suggesting a compensatory mechanism between the growth in length and in thickness in maize root, highlighted the specificity of nitrate in affecting the root growth, which conversely did not show any similar response when nitrogen was supplied as ammonium.

### NR-dependent NO production after nitrate supply

**Fig. 5.**
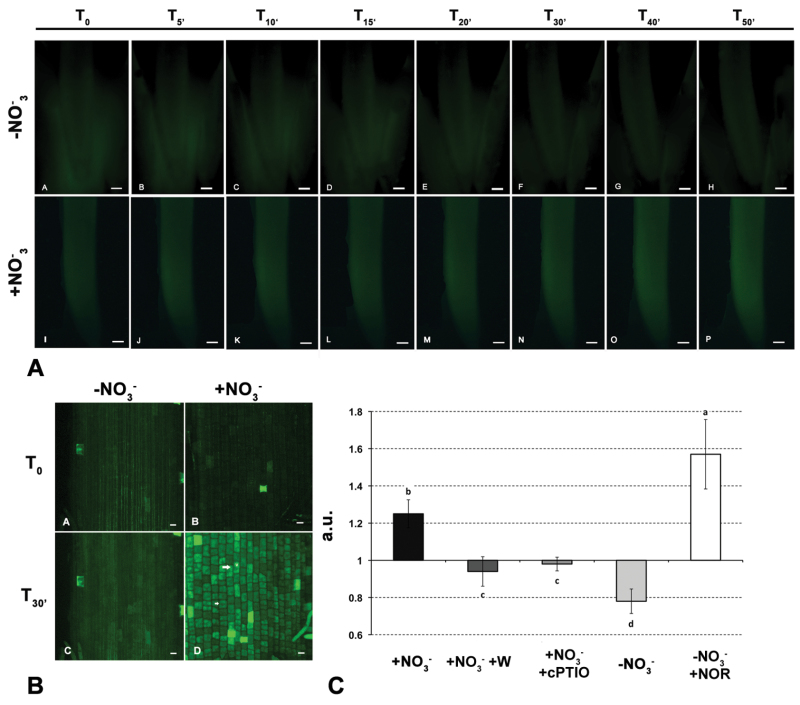
NO detection on 2-cm maize root apices excised from seedlings grown for 24h in nitrogen-depleted nutrient solution. (A) Stereomicroscope time course imaging of DAF-2T fluorescence (T_0_–T_50_) on apices treated for 30min with 1mM KCl (negative control, –NO_3_^–^) (A–H), and 1mM KNO_3_ solution (I–P); bars, 500 µm. (B) Confocal detection of DAF-2T in the transition zone of nitrate-treated and untreated apices at T_0_ and T_30_. Arrows indicate two different types of cells of this root zone: small square-shape cells with central nucleus and elongated cells with a more developed vacuole (V); bars, 50 µm. (C) DAF-2T fluorescence intensity values at 30min after treatment of root segments with NO_3_^–^, NO_3_^–^ and tungstate (W), NO_3_^–^ with the NO scavenger cPTIO, KCl (–NO_3_^–^), KCl (–NO_3_^–^) and NO donor NOR. Mean fluorescence values are reported as a ratio of the fluorescence intensity at 30min to the fluorescence intensity at time 0 (arbitrary units, a.u.). Different letters indicate statistically significant differences between treatments (*P* < 0.05, Kruskal–Wallis test) (this figure is available in colour at *JXB* online).

To better understand the role of NO in nitrate signalling, its production was monitored by measuring the DAF-2T fluorescence in stereomicroscopy. Seedlings grown for 24h without nitrate were supplied with 1mM nitrate and the fluorescence produced was observed (Fig. 5A, panels I–P) in comparison with that measured in negative controls (Fig. 5A, panels A–H). The nitrate supply caused a slight but consistent increase in DAF fluorescence in the first minutes after treatment (panels J and K). No fluorescence increase was induced by NO_3_^–^-deprived control treatments, whereby in contrast a signal decrease was observed after 10min, probably due to the decay of the probe (panels A–H). Based on these observations, the increment of fluorescence was mainly localized immediately above the meristematic apex and more precisely in the transition zone, as defined by Verbelen *et al.* (2006) and Mugnai *et al.* (2012).

In order to get a more detailed imaging and quantification of DAF fluorescence, the experiment was repeated at the confocal microscope and the effects of a NO donor (NOR), a NO scavenger (cPTIO), and a NR inhibitor (tungstate) were evaluated. Results for both –NO_3_^–^ and +NO_3_^–^ treatments at T_0_ and after 30min of observation are shown in Fig. 5B. There is a clear difference between the two treatments, with a strong increase in the DAF fluorescence in response to nitrate provision (panel D) that was not observed in negative controls (–NO_3_^–^ roots, panel C). Moreover, higher-magnification analyses (Supplementary Fig. S3) revealed a few cytological details on the different cell types observed, which typically distinguish the transition zone. In the distal part of the portion of root examined, nuclei were positioned in the centre of the cell, similarly to the meristem, whereas the more distal zone cells resembled those of the elongation zone with large central vacuoles and nuclei pushed to the side cell walls. The same observations were performed in the presence of tungstate, NOR, and cPTIO. The results obtained with five biological repetitions are reported in Fig. 5C. Data were expressed as relative fluorescence increase/decrease after 30min of observation. Results showed a significant increase of fluorescence for nitrate-supplied and NOR-treated roots. On the contrary, when seedlings were supplied with –NO_3_^–^-solution (negative control), with nitrate plus tungstate, or nitrate plus cPTIO, the fluorescence did not increase throughout the experiment. These results suggest that a NR-dependent NO burst occurred immediately after nitrate supply to roots.

### Genes putatively involved in the control of NO homeostasis participate in the early response to nitrate

Due to the size of the mini-chamber utilized for both stereo- and confocal microscopy, it was necessary to work with roots sampled from younger seedlings. For this reason, we shifted the experimental plan to younger seedlings also for the following expression analyses, focusing only on the early events after nitrate provision. Plants were, thus, grown for 24h in –NO_3_^–^ solution and then transferred to +NO_3_^–^ medium for 2h. The transcript accumulation of the previously selected genes (*NR1*, *Hb*, *Hb2*, *NiR*) together with a new one (*NOA1*) encoding a putative *AtNOA1* orthologue was observed after nitrate supply and in the presence of cPTIO, tungstate, or l-NAME. The expression of *NRT2.1* was also included among the analyses as a positive control of the nitrate perception.

**Fig. 6.**
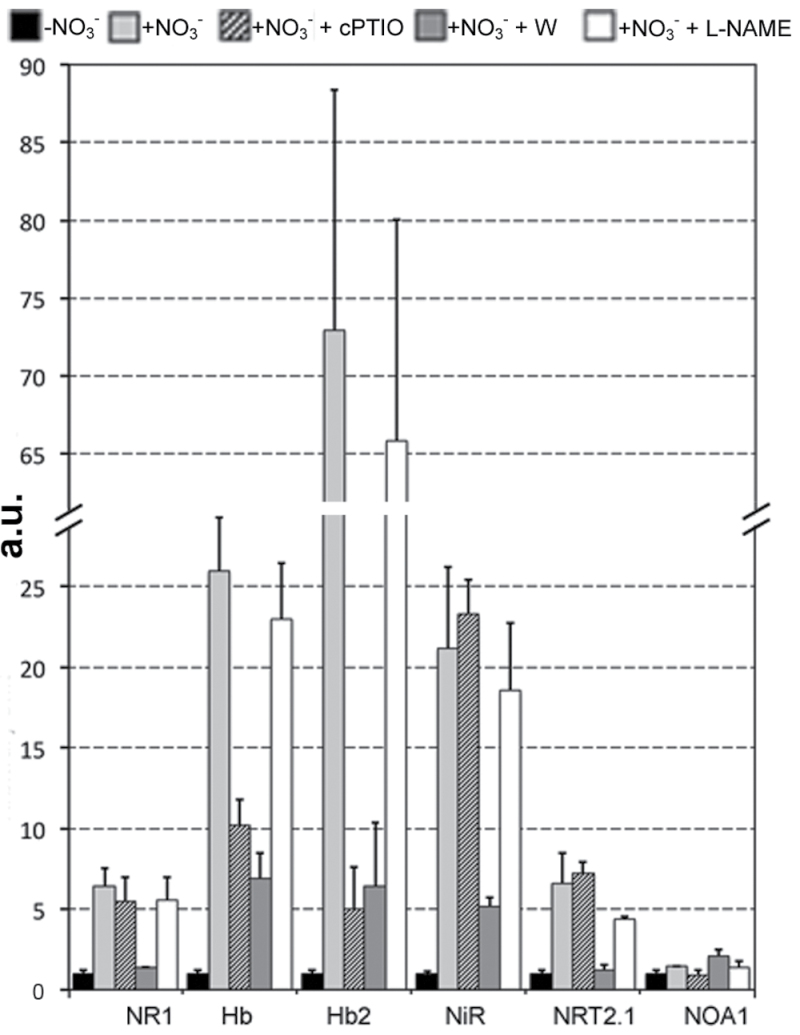
Effects of five different chemicals interfering with NO biosynthesis and scavenging on the expression profile of six genes differentially regulated by nitrate supply/depletion. Plants were grown for 24h in –NO_3_^–^ solution and then transferred to +NO_3_^–^ medium for 2h. The transcript accumulation of six genes (*NR1*, *Hb*, *Hb2*, *NiR*, *NRT2.1*, and *NOA1*) was examined after nitrate supply and in the presence of 1mM cPTIO, 1mM tungstate (W), and 0.2mM l-NAME. a.u., arbitrary units.

Nitrate addition induced strong increments of transcription for all genes analysed except for *NOA1* (Fig. 6, first two columns for each gene). The expression of NR gene was 6–9-fold higher in comparison to that measured in –NO_3_^–^ roots, whereas the two isoforms of nsHb increased their transcription by 27–72-fold. *NiR* and *NRT2.*1 showed an induction of their expression of 21- and 6-fold, respectively. When cPTIO was given together with nitrate (third column), the expression of both *Hb* and *Hb2* was very strongly inhibited, whereas the other genes analysed did not evidence significant differences of expression in comparison to the positive control (+NO_3_^–^). Similarly, the addition of tungstate (fourth column), led to an inhibition of 75–90% of transcription of all genes, with the exception of *NOA1*. Conversely, the provision of l-NAME, an inhibitor of nitric oxide synthase (NOS), induced only slight and rarely significant decreases of the expression of these genes.

These results confirmed the role of the regulation of *NR1*, *Hb*, and *NiR* in the early response to nitrate even in younger roots. Moreover, the use of chemicals interfering with NO biosynthesis and scavenging provided further evidence of the involvement of NR-derived NO as a key signal in nitrate signalling in roots of maize.

### Transcription of genes involved in NO production and scavenging is maximally induced in root transition zones after nitrate induction

**Fig. 7.**
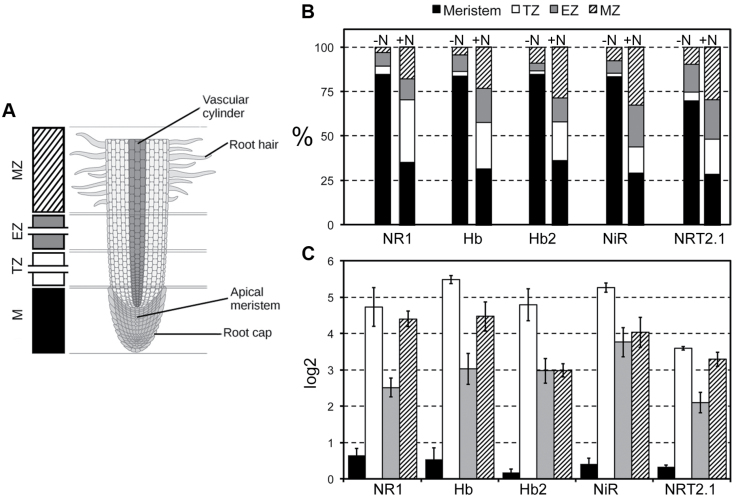
Spatial distribution of five genes differentially regulated by supply/depletion of nitrate. (A) Graphical representation of the different parts of primary root analysed; M, meristem; TZ, transition zone; EZ, elongation zone; MZ, maturation zone. (B) Gene expression in the different zones reported as percentage in both nitrate-starved and -supplied roots; percentages are expressed as the ratio between the mRNA abundance measured in each specific root zone and the global amount of transcript in the overall root. (C) Increase of transcription for each gene in each of the four portions; data are as log_2_ of the ratio of +N/–N.

Results on NO measurements suggest that the production of this molecule after nitrate provision is preferably localized immediately above the meristematic apex, and more precisely at in the transition and elongation zones. The expression of the genes encoding nitrate reductases, haemoglobins, and nitrite reductase and of *NRT2.1* was, therefore, studied in four different root portions (meristem, transition zone, elongation zone, maturation zone, as schematized in Fig. 7A), both in nitrate-depleted roots and after 2h of nitrate provision. All five genes evidenced a significant change of localization when seedlings grown without nitrate were treated with the anion (Fig. 7B). In fact, in nitrate-starved roots, 70–80% of mRNA was concentrated in the meristematic cells for all five genes, with the remaining 20–30% of mRNA prevalently localized in the elongation and maturation zones. In these conditions, the amount of transcript detected at the transition zone level was extremely low or even negligible. On the contrary, in seedlings supplied with 1mM nitrate for 2h (after being grown 24h in –NO_3_^–^ solution), the transcripts of all five genes were more equally distributed between the apical meristem and the transition zone, with a significant increase of accumulation in the transition zone which showed an amount of mRNA for each gene ranging from 20 to 40% of the total. Moreover, after nitrate supply the maturation zone also evidenced an increase in terms of gene expression compared with nitrate-depleted roots.

Fig. 7C describes the increases of transcription for each gene in each of the four portions, independently from their relative abundance. All five genes evidenced an induction of their expression in all the four portions sampled, with the maximum in the transition zone which showed a transcription rate more than 30-times higher if compared with that measured in the same portion of nitrate-depleted roots (except the case of *NRT2.1*, which increased more than 10-times). In the elongation and maturation zones of nitrate-supplied roots, the amount of mRNA increased by around 4–8 and 8–20 times, respectively. On the contrary, in the meristematic cells the increase of gene transcription measured was very low or insignificant.

In general, it would seem that nitrate supply induces a redistribution of transcripts in zones of roots different from the meristem, which in turns appears to be the main site of their accumulation in conditions of nitrate starvation.

### The nitrate-induced root length increase is dependent on a NO signalling pathway

**Fig. 8.**
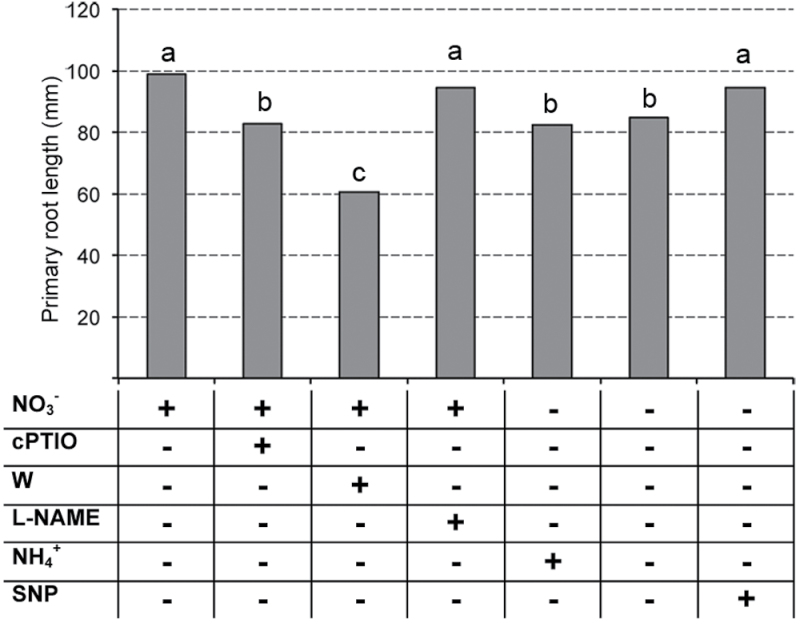
Effect of different nitrate treatments on primary root growth. After germination seedlings were transferred to different nutrient solutions (+NO_3_^–^, –NO_3_^–^, +NH_4_^+^, +NO_3_^–^ +W,–NO_3_^–^ +SNP, +NO_3_^–^ +cPTIO, +NO_3_^–^ + l-NAME) and the growth of primary root was measured for 24h with a ruler on 16 seedlings for each group. Values represent the mean of four independent biological repetitions. Different letters indicate significantly different means (*P* < 0.05).

After germination, seedlings were transferred to seven different nutrient solutions (+NO_3_^–^, –NO_3_^–^, +NH_4_^+^, +NO_3_^–^ +tungstate, –NO_3_^–^ +SNP, +NO_3_^–^ +cPTIO, +NO_3_^–^ +l-NAME) and the growth of primary root was monitored for 24h (Fig. 8). Nitrate-supplied seedlings and –NO_3_^–^ +SNP seedlings showed a more elevated rate of root elongation, with values significantly higher in comparison to all the remaining treatments. The supply of ammonium did not produce any increase in the elongation rate, which was similar to that measured for negative control plants, as already observed also for older seedlings. The provision of tungstate together with nitrate inhibited even more significantly the root growth in comparison with nitrate-depleted roots. A similar decrease was also observed in roots supplied with nitrate plus cPTIO. On the contrary, the addition of SNP to nitrate-depleted roots stimulated root growth to levels comparable to those measured for positive control. Conversely, as also observed in the case of gene transcription, the supply of l-NAME, which inhibits NOS activity, did not produce significant effects on root lengthening.

These results, besides suggesting the involvement of NO in the regulation of nitrate-induced root elongation, clearly confirm the key role of NR for this signalling pathway.

The fresh weight of both roots and shoots was also determined to exclude toxicity effects of chemicals utilized (Supplementary Table S2).

### The nitrate-induced NO signalling pathway is a localized effect

The set up of a method to grow maize seedlings on a semisolid agar medium allowed to perform targeted treatments to single zone of root (Fig. 2). This system permits the treatment of only specific zones of root, thus enabling the discrimination between local and systemic effects on gene expression.

**Fig. 9.**
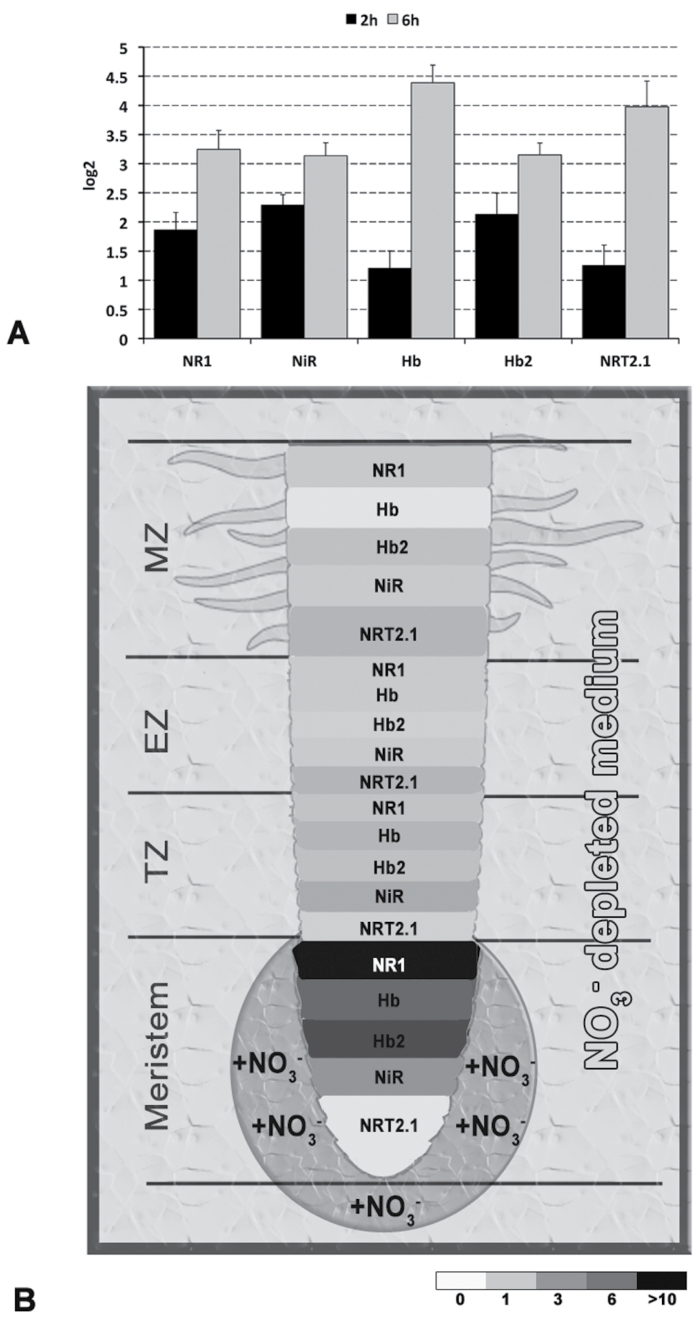
Expression analysis of NO- and nitrate-metabolism-related genes (*NR1*, *Hb*, *Hb2*, *NiR*, and *NRT2.1*) on roots of 24-h-old seedlings grown on nitrate-depleted agar and treated in a fresh medium added with nitrate in the whole plate or locally at the root tip. (A) Roots at 2h (black bars) and 6h (grey bars) after treatment; data are expressed as log2 ratios of the normalized expression levels measured in treated roots with respect to the control (no nitrate) grown at the same conditions; values are means ± SE of six independent biological replicates, each performed in three technical repetitions. (B) Fold-change (reference: untreated meristem) in gene expression along the root treated locally at the meristem zone with nitrate. The sizes of the different zones do not reflect the real values of length; please refer to the Material and methods for the exact measures. Values of fold-change are expressed by means of a grey scale. The authors arbitrarily chose the size of each block occupied by single gene, which do not reflect any quantitative value. M, meristem; TZ, transition zone; EZ, elongation zone; MZ, maturation zone.

As a preliminary experiment, to test the validity of this method as an alternative to hydroponics, seedlings were grown on nitrate-supplied or nitrate-deprived agar, using the same timing and concentrations as for the experiments in hydroponics, and the expression of the previously selected genes was evaluated. RT-qPCR were carried out on both roots and shoots and for all the genes and the nutritional conditions described in the first paragraph of the Results (data not shown). Figure 9A shows only the genes closely related to the induction of NO pathway in roots after nitrate supply. The results fully confirmed those obtained for seedlings grown in hydroponics. Furthermore, the root growth, analysed by means of WinRhizo software, evidenced the same behaviour of plants grown in nutrient solution (Supplementary Fig. S4), further confirming the validity of this method.

Seedlings were then submitted to a treatment with nitrate localized only to the meristematic apex (4mm). The transcription of the five genes previously chosen was evaluated independently in the four root zones (meristem, transition zone, elongation zone, maturation zone) in both seedlings whose tips was treated with 1mM nitrate and negative control (Fig. 9B). *NR1*, *Hb1*, and *Hb2* strongly increased their expression in the apices of nitrate-supplied roots, whereas *NiR* transcription increased to a lesser extent. On the contrary, abundance of *NRT2.1* mRNA did not evidence any increase, indicating that this high-affinity nitrate transporter is not involved in the influx of nitrate by the root meristem. Furthermore, in the three other root zones, no differences in terms of transcript accumulation were detected after local nitrate provision to the root apex, suggesting that NO signalling activation by nitrate should represent a localized effect of nitrate.

## Discussion

Nitrogen is a major element for plant life, and crops strongly depend on intense fertilization programmes throughout the world, thus affecting environment quality. The identification of crop cultivars with improved nutrient acquisition efficiency in low-input farming systems continues to be a real priority for plant scientists (Robertson and Vitousek, 2009; Xu *et al.*, 2012). Nitrate is the main nitrogen source for plants in regular agricultural systems and, acting also as a signal, triggers a number of molecular and physiological events leading to the overall plant’s response to its availability (Gojon *et al.*, 2010 and references therein).

The control of NO homeostasis through the spatiotemporal coordination of nitrate reductase and haemoglobin gene expression has been recently hypothesized to participate to nitrate sensing in maize roots (Trevisan *et al.*, 2011). The present work tried to more deeply characterize the role of NO in the maize root response and adaptation to nitrate fluctuations.

To better discriminate nitrate-specific effects from those more generally N-dependent, the expression of previously selected genes (Quaggiotti *et al.*, 2003; Trevisan *et al.*, 2011, 2012) was evaluated in response to nitrate or ammonium supply and deprivation (Fig. 3). This first screening allowed the current work to focus later only on genes responding exclusively to nitrate (and not to ammonium), which coincided with those involved in the control of NO biosynthesis and scavenging. In particular, genes encoding the cytosolic NR and two different nsHbs, together with a gene encoding nitrite reductase, evidenced both in short-term and long-term experiments a clear and noticeable responsiveness to nitrate supply or starvation, but did not change their expression in response to ammonium (Figs 3 and 4). A gene encoding a high-affinity root nitrate transporter was also used as internal control, in light of its putative role in nitrate influx and its transcriptional inducibility during the first phases of nitrate supply (Quaggiotti *et al.*, 2003). The expression profile recovered for this gene provided indirect evidence of the entry of nitrate into root epidermal cells, hence enabling the activation of the signalling pathways in which nitrate is involved.

Besides being the first enzyme of nitrate assimilation, NR represents also one of the most important sources of NO in plants (Mur *et al.*, 2012). It is a cytosolic enzyme that can reduce both nitrate to nitrite and nitrite to NO, even if it shows a better affinity for nitrate than for nitrite. However, NR seems to be switched to the latter reaction when high nitrite levels are produced (Gupta *et al.*, 2011; Mur *et al.*, 2012). This occurs, for example, when the external nitrate rapidly increases after a nitrate starvation, as a consequence leading to a strong increase of NO_3_^–^ influx inside cells, as it might have happened in the current study. Once inside the root cells, nitrate is promptly converted to nitrite by NR, leading to nitrite accumulation. Nitrite accumulation could in turn shift the NR equilibrium toward its second mode of action, thus promoting the biosynthesis of NO in response to nitrate. This scenario seems consistent with the main findings of this paper. Nitrate reductase is involved in NO production during bacteria-induced defence (Modolo *et al.*, 2005), disease development in certain pathogenic interactions (Shi and Li, 2008), drought (Freschi *et al.*, 2010), cold (Zhao *et al.*, 2009), osmotic stress response in roots of *Arabidopsis* (Kolbert *et al.*, 2010), stomatal regulation (Srivastava *et al.*, 2009), and many developmental processes such as the initiation of flowering (Seligman *et al.*, 2008).

The strong increase of the expression of both of the *nsHbs* genes observed after 30min of nitrate supply is not surprising if considering the high reactivity of NO which, as well as serving as a signal in regulating several physiological events, must also be kept at a steady level to avoid damage due to its toxicity. Recently, several studies have indicated a role for haemoglobins in detoxification of high intracellular NO concentrations (Dordas *et al.*, 2003*a*,*b*; Perazzolli *et al.*, 2004; Vieweg *et al.*, 2005). The patterns of expression of non-symbiotic haemoglobins vary depending on tissues and in response to different types of stress (Hunt *et al.*, 2001). Perazzolli *et al.* (2004) provided evidence that *Arabidopsis* non-symbiotic haemoglobin AtHb1 functions as a NO-dioxygenase, metabolizing NO to nitrate. Moreover, plant haemoglobins seem to be involved in the control of NO accumulation during rhizobial and mycorrhizal symbioses (Vieweg *et al.*, 2005) and in the response to hypoxia in different tissues such as seeds, roots, and stem tissue (Dordas *et al.*, 2003a,*b*). Plant haemoglobins can control developmental and physiological reactions by modulating cellular NO levels (Hill, 2012) and should be considered to be as important as NO generation in regulating *in planta* NO signalling (Mur *et al.*, 2012).

These results, besides confirming the already hypothesized involvement of NO homeostasis control in the maize root response to nitrogen (Trevisan *et al.*, 2011), demonstrate also that this is an exclusive prerogative of NO_3_^–^ signalling. In fact, when ammonium was supplied to nutrient solution as the sole nitrogen source, no significant effects were measured on the transcription of genes involved in NO production and scavenging. On the contrary, the expression of the other genes here analysed did not show a similar nitrate-specific responsiveness. In addition, data obtained by analysing root morphological parameters by the WinRhizo software highlighted the same specificity of nitrate, which significantly affected root growth when supplied to N-deprived roots, in contrast to what happened when the same concentration of ammonium was given to roots (Table 1).

Thus, it would seem that NO may be produced by roots as an early signal of nitrate perception. To deepen this hypothesis, *in vivo* NO detection was carried out. Results obtained using the DAF-2DA probe (Kojima *et al.*, 1998) and stereo- and confocal microscopy evidenced a clear induction of fluorescence after nitrate provision (Fig. 5). The main zone of NO production seemed to be located immediately above the meristematic apex. A similar localization has been recently observed in the same species by Mugnai *et al.* (2012) as a response to hypoxic conditions. The fluorescence detected after nitrate supply was not relieved in the presence of tungstate, giving support to the role of NR in this process. Moreover, the addition of cPTIO suppressed the development of fluorescence, confirming the specificity of NO detection by the probe. This was also corroborated by the strong increase of fluorescence when NOR was supplied to nitrate-depleted roots.

To give more strength to these results, this work tried to operate by following the steps indicated by Mur *et al.* (2012), being well conscious that it should be always preferable to employ parallel approaches for NO measurements. The NR-dependent NO production observed after nitrate supply was then corroborated by the expression analyses performed on roots of 1-day-old seedlings (Fig. 6). In particular, the results proved the strong induction of *NR1*, *NiR*, and *nsHbs* transcription in the early phases of nitrate perception. As also observed in the case of NO production, the transcription of all genes was significantly inhibited after tungstate and cPTIO addition, confirming the cooperation between nitrate reductase and haemoglobin activities in the finely tuned control of NO homeostasis. However, to exclude the possible involvement of sources of NO other than NR, the study was extended also to the orthologous gene of *Arabidopsis*, *AtNOA1* (Guo *et al.*, 2003), encoding the nitric oxide associated 1 protein (Zemojtel *et al.*, 2006). NOA1 was previously named AtNOS1 and it has been described as a potential NOS in *A. thaliana*, despite lack of sequence similarity to animal NOSs. It has been successively established to be a GTPase (Moreau *et al.*, 2008) and not to possess NOS activity, and for this reason it has been renamed AtNOA1. Previous studies have shown that NOA1-dependent NO synthesis is involved in hormonal signalling, stomatal movement, flowering, pathogen defence, and oxidative stress (Guo *et al.*, 2003; He *et al.*, 2004; Zeidler *et al.*, 2004; Zhao *et al.*, 2007). The transcription of the orthologous *AtNOA1* in maize did not evidence any alteration neither in response to nitrate nor to the other chemicals utilized.

Moreover, to exclude the involvement of a more generic NOS activity, nitrate-supplied seedlings were also treated with l-NAME, which is commonly used to inhibit NOS activity in mammalians and also in plants. No effects, neither on transcription of nitrate-responsive genes (especially with regards to *nsHbs*) nor on the nitrate-induced root lengthening, were observed (Figs 6 and 8), giving more strength to the idea that NO production after nitrate provision is predominantly dependent on the activity of NR.

To deepen the spatial regulation of NO homeostasis balance, the expression of the five genes was analysed in four different root zones (meristem, transition zone, elongation zone, maturation zone) both in nitrate-depleted and in nitrate-treated (1mM) seedlings (Fig. 7). In N-starved roots, all five transcripts evidenced their maximum accumulation at the meristem level. This pattern radically changed when nitrate was furnished to roots with a very significant increase of transcript abundance in the transition zone, located between the meristem and the region of fast cell elongation. Cells of the transition zone undergo a series of fundamental changes in their cytoarchitecture and physiology and they accomplish dramatic rearrangements of the actin cytoskeleton (Baluška *et al.*, 1997, 2001). This is essential for the developmental switch into rapidly elongating root cells which expand strictly uniaxially (Baluška *et al.*, 1997). The distal part of this zone in characterized by a prevalence of cells that optionally can re-enter the cell cycle, whereas the proximal part is equipped with cells competent to rapidly enter the fast cell elongation zone. As this developmental passage of cells can be differentially regulated at the opposite root flanks, this unique zone provides the root apices with an effective mechanism to reorientate growth in response to environmental stimuli (Verbelen *et al.*, 2006). A number of experimental proofs suggest that the transition zone should be considered as a sort of sensory and information processing zone, enabling the growing root apex to monitor environmental parameters continuously and to trigger appropriate responses (Mugnai *et al.*, 2012). If this is true and if a role for NO homeostasis control through the combined action of NR and nsHb in the early perception of nitrate by roots is hypothesized, the current results on transcript accumulation redistribution along root apex are not surprising. Based on this finding, it would seem that nitrate supply could activate its own sensing by stimulating NO production by the transition zone cells, thus initiating a signalling pathway contributing to the physiological adaptation (e.g. root growth) to nitrate fluctuations.

The most important example of the plasticity that plants express to fit with nutrient withdrawal in soil is, in fact, represented by the capability of rearranging root architecture to maximize their capture (Lόpez-Bucio *et al.*, 2003; Hermans *et al.*, 2006; Zhang *et al.*, 2007; Zolla *et al.*, 2010; Giehl *et al.*, 2012; De Pessemier *et al.*, 2013). Nitrate affects root development by finely regulating the growth of lateral roots depending on its external concentration and localization, as aforementioned (Péret *et al.*, 2009; Mounier *et al.*, 2013; Yu *et al.*, 2013) and as also showed by the current findings obtained with the WinRhizo software (Table 1). Based on the preliminary results showing the preferential localization of NO production at the level of the transition zone, the attention was focused on nitrate effects on root elongation, which takes place in the zone immediately above and neighbouring the transition zone (Fig. 8). The results evidenced a strong and specific induction of root elongation of young maize seedlings supplied with 1mM nitrate and a drastic inhibition in the presence of ammonium, cPTIO, and tungstate. No effects were recorded in the presence of l-NAME. On the contrary, when the negative control (–NO_3_^–^) was supplied with a NO donor (SNP) the root length increased significantly. These results strongly suggest that the NO generated through NR should significantly contribute to the root lengthening noticed after nitrate provision.

The involvement of NO in root development has been observed in numerous studies, such as those published by the Lamattina group (Pagnussat *et al.*, 2002, 2003; Correa-Aragunde *et al.*, 2004; Lombardo *et al.*, 2006), but it was already being hypothesized in 1997 by Gouêva *et al*., who found that NO was able to induce cell elongation in a way similar to auxin. Moreover, a recent study suggested that class-2 non-symbiotic haemoglobins play a role in regulating the synthesis and transport of auxins by altering the level of the signal molecule, NO, in specific cells (Elhiti *et al.*, 2013). Besides this, NO is involved in the regulation of actin cytoskeleton, endocytosis, vesicle trafficking, and the polarity of growing tip cells (Prado *et al.*, 2004; Lombardo *et al.*, 2006; Salmi *et al.*, 2007; Prado *et al.*, 2008; Kasprowicz *et al.*, 2009; Wang *et al.*, 2009), which are all prerequisites to acquire competence for cells to elongate. Considering also that NO is widely implicated in the plant response to environmental stresses (Beligni and Lamattina, 2001; Dat *et al.*, 2004), it seems to play crucial functions at the crossroads between developmental and abiotic stress tolerance. For this reason, it should also represent a very good molecular candidate to regulate root development in response to abiotic stresses, such as nutrient or oxygen deprivation (Mugnai *et al.*, 2012), but also an early player in symbiotic interactions establishment, which also need root architecture to be adapted to the environment.

In the present research, thanks to a method allowing the growth of maize seedlings in vertical plates with an agar medium, some major details on NO-mediated nitrate signalling have been attained. The results suggest that the mechanism underling the root response to nitrate and involving NO signalling is directly activated on cells which enter in contact with external nitrate (Fig. 9). Moreover, this alert system does not seem to be turned on by some nitrate-derived compounds or by the nitrate that moves up through the root. In fact, when only the meristematic apex was treated with nitrate, the induction of the transcription of *NR1* and *Hb*s was exclusively restricted to the apex itself, whereas in the upper zone of the roots no differences were detected in comparison with the negative control. This is even more remarkabe considering that, conversely, when the entire root is in contact with nitrate, the apex is the portion that shows the lower responsiveness to this anion in terms of induction of gene expression, the transition zone being the most receptive. Moreover, these results indicate that nitrate transporters other than NRT2.1 should be implicated in nitrate perception at the root meristem, since the transcription of *NRT2.1* is not activated at all by nitrate, in contrast to what was observed in all the other three root zones when the whole root was supplied with nitrate.

**Fig. 10.**
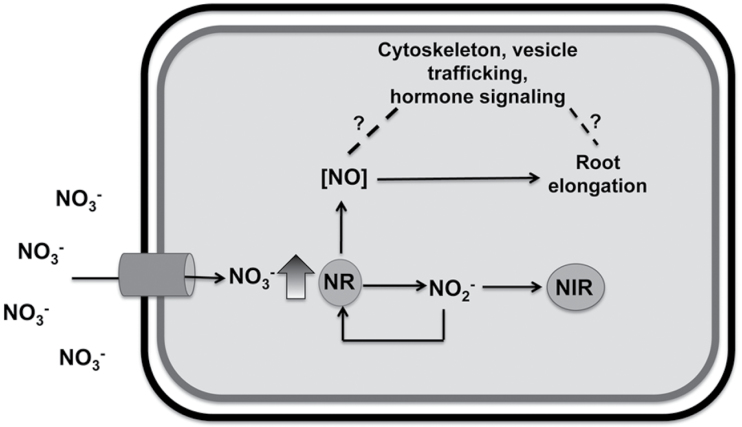
Model for the NO-mediated nitrate induction of root elongation. NO_3_^–^ influx is performed by specific nitrate transporters (e.g. NRT2.1 in the TZ, EZ, and MZ). Once inside the root cells, NO_3_^–^ is able to act as a signal to induce its own sensing via the NR/Hb-dependent NO fine-tuning, which in turns seems to be involved in the root elongation stimulation. The cytological events and molecular targets linking the NO biosynthesis to root growth response could be involved in the rearrangements of the actin cytoskeleton (Baluška *et al.*, 2001) and need to be further studied and characterized.

Based on these data, it would seem that the NO-mediated pathway here described represents an early alert system for external nitrate sensing by root cells, which seem to individually possess the competence to activate this pathway when external nitrate is perceived. But also, since root growth is modulated by the convergence of multiple environmental inputs which are integrated by specific signal pathways to decide how to explore the surrounding environment, additional experiments will be needed to better understand the functioning of this NO-mediated pathway and to identify the downstream events that link the NO burst with the physiological redirection of root growth. Even if a high number of specific and comprehensive issues on the NO role in the complicated cross point between root and nitrate (and root and abiotic stress perception in general) need to be further deepened, the current findings suggest that the triggering of a NO burst is a direct response to the rapid increase of nitrate availability and that it could mediate the root elongation observed after nitrate provision (Fig. 10).

## Supplementary material

Supplementary data are available at *JXB* online.

Supplementary Fig. S1. The improved agar-plate culture system for studying the *Zea mays* L. root response to different nutrient availability.

Supplementary Fig. S2. Time course of the expression of genes following short-term nitrate/ammonium treatments in maize roots and leaves.

Supplementary Fig. S3. Confocal detection of DAF-2T in the transition zone of nitrate-treated apices.

Supplementary Fig. S4. Root and leaf fresh weight and relative root/shoot ratio in seedlings grown in nutrient solution for 5 days and total root length, total surface area, mean diameter, number of root tips, and leaf fresh weight in seedlings grown for 5 days in agar with or without 1mM NO_3_^–^.

Supplementary Table S1. List of genes analysed by real-time quantitative PCR.

Supplementary Table S2. Merged effects of different chemicals interfering with NO biosynthesis/scavenging and nitrate supply/depletion on root and leaf fresh weight.

Supplementary Data
