# Supplementary material for: NO homeostasis is a key regulator of early nitrate perception and root elongation in maize
Source: J Exp Bot. 2013 Nov 12;65(1):185–200. doi: 10.1093/jxb/ert358 (PMC3883287; doi:10.1093/jxb/ert358)
Supplement: Supplementary Data [file supp_65_1_185__index.html]

NO homeostasis is a key regulator of early nitrate perception and root elongation in maize — NO homeostasis is a key regulator of early nitrate perception and root elongation in maize — Supplementary Data 

# NO homeostasis is a key regulator of early nitrate perception and root elongation in maize

## Supplementary Data

Data files

**Files in this Data Supplement:**

- Supplementary Data - Supplementary Data
