# Supplementary material for: NO homeostasis is a key regulator of early nitrate perception and root elongation in maize
Source: J Exp Bot. 2013 Nov 12;65(1):185–200. doi: 10.1093/jxb/ert358 (PMC3883287; doi:10.1093/jxb/ert358)
Supplement: Supplementary Data [file supp_ert358_jexbot105601_file001.pdf]

NO homeostasis is a key regulator of early nitrate perception and root elongation in maize

Manoli Alessandro, Begheldo Maura, Genre Andrea, Lanfranco Luisa, Trevisan Sara and Quaggiotti Silvia

Supplementary data

Figure S1
The new improved agar-plate culture system for studying the *Zea mays* L. root response to different nutrients availability.

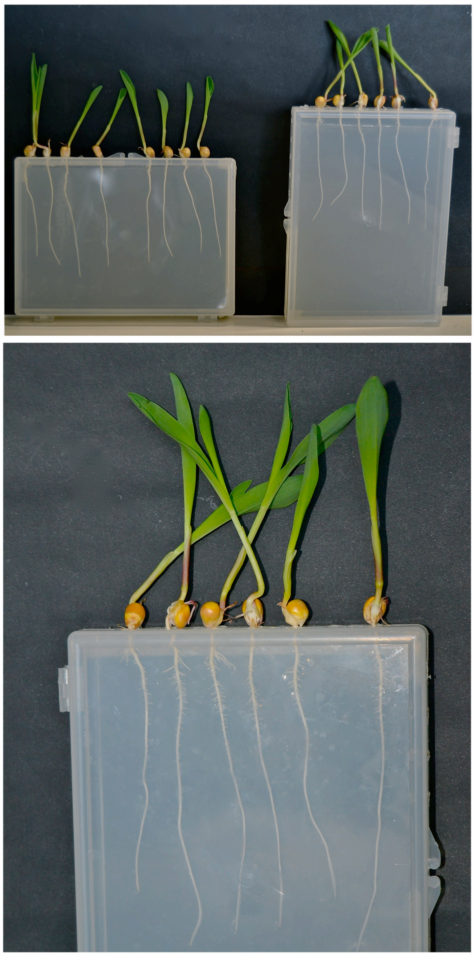

Figure S2

Time course of the expression of genes following short-term nitrate/ammonium treatments in maize roots (A) and leaves (B). The expression values were investigated at 30 min, 2 h and 6 h.

Data are expressed as base-2 logarithm of the ratio between the expression measured for a treatment and that of its own control. The same expression analyses were carried out in nutrient solution either supplied or depleted with nitrate or ammonium.

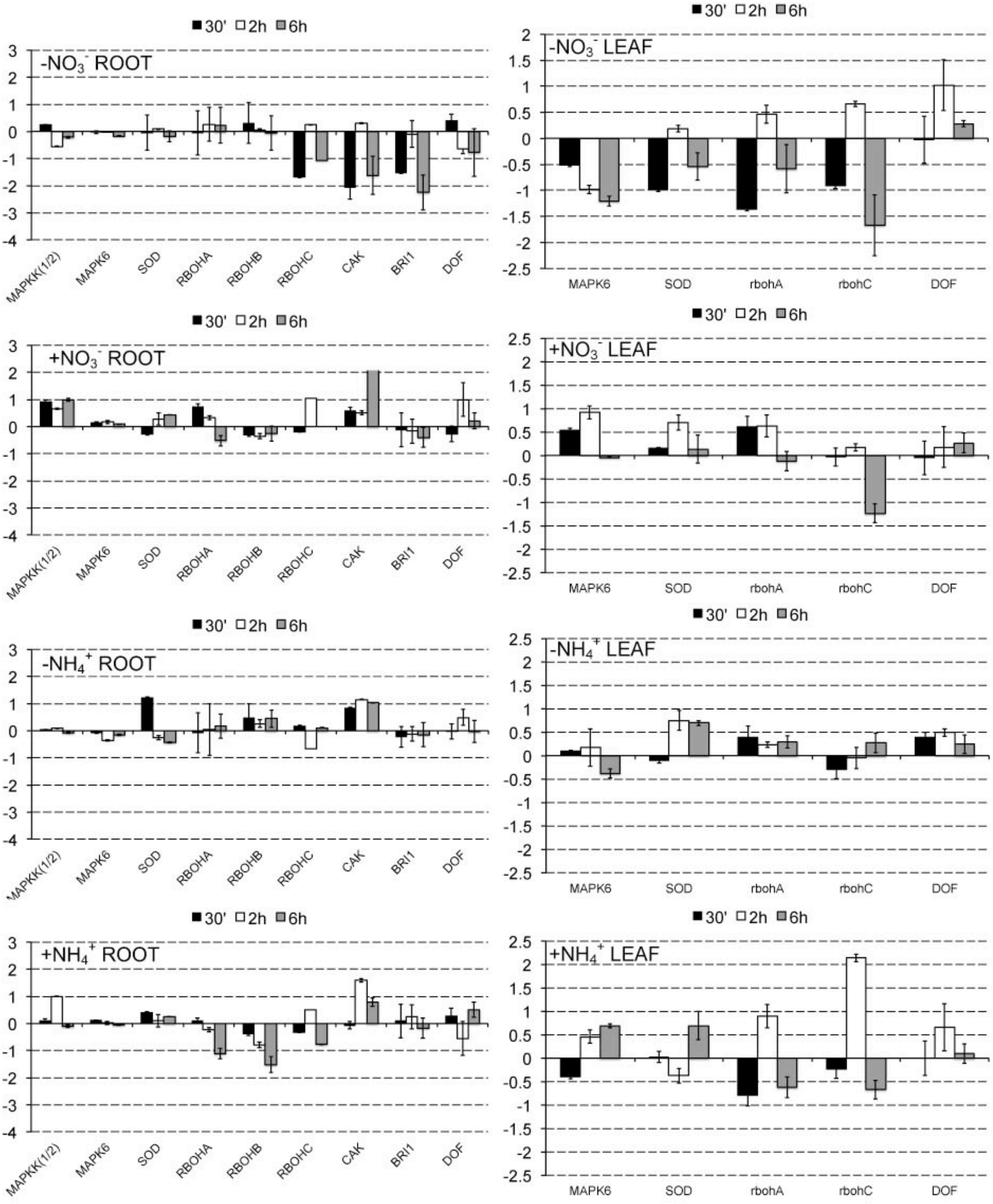

Figure S3

Confocal detection of DAF-2T in the transition zone of nitrate treated apices. Arrows indicate two different types of cells of this root zone: small square shape cells (red arrows) with central nucleus (N) and elongated cells (white arrows) with a more developed vacuole (V). Image was obtained by zooming the 40x lens.

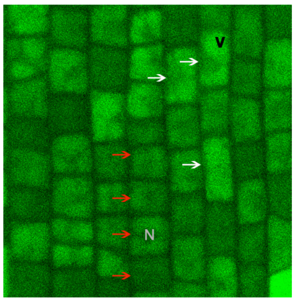

Figure S4

Root and leaf fresh weight and relative root/shoot ratio in seedlings grown in nutrient solution for five days (A, B, C).

Total root length (L), total surface area (SA), average diameter (AD), number of root tips and leaf fresh weight in seedlings grown five days in agar medium containing or not 1 mM NO3- (D).

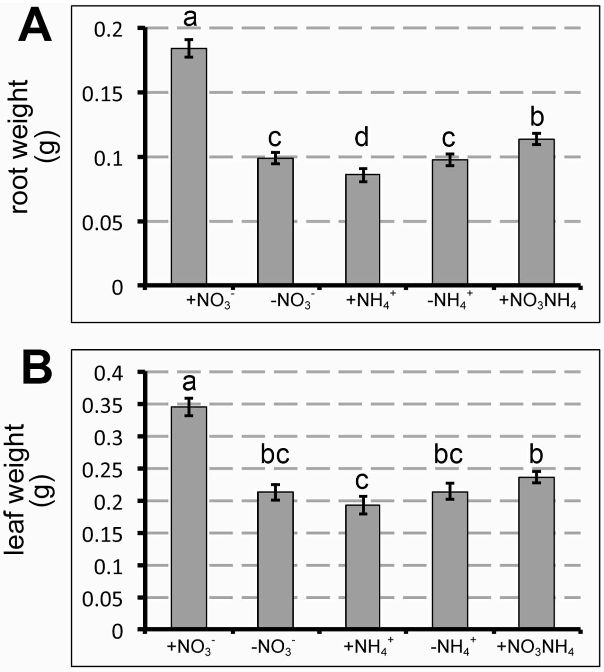

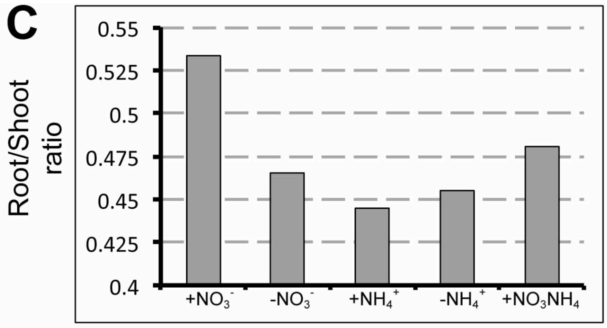

D

	L (cm)	SA (cm^2)	AD (mm)	Tips (n)	leaf weight (g)
**+N**	27,6	5,14	0,65	58	0,13
**-N**	22,5	4,18	0,61	43,6	0,10
ANOVA p<0.05	*	*	ns	**	**

Table S1

List of the genes analyzed by means of Real Time qPCR. Maize GDB and NCBI accessions are reported together with the gene functions and the primer sequences.

Maize GDB Accession ID	NCBI	Name	Gene Function	Fw	Rv
GRMZM2G067402 T02	AF236080.1	Hb	haemoglobin	GGAGCCTCGAGATGAAGAAA	ACAATACACGCTCCCTCCAG
GRMZM2G168898_T01	NM_001112349.1	Hb2	haemoglobin 2	GGCTGTTGATGCTTCCTAGC	ATGACGGGCCTTTTCTGAAT
GRMZM2G568636	AF153448.1	NR1	nitrate reductase	ATGATCCAGTTCGCCATCTC	GTCCGTGGTACGTCGTAGGT
GRMZM2G079381	EU957616.1	NiR	ferredoxin--nitrite reductase	CTTCATGGGCTGCCTCAC	CGCTTGACGAAGGTCCTACT
GRMZM2G010280 T01	AY129953.1	NRT2.1	putative high affinity nitrate transporter	GAGAAGAGCAAGGGACTCCA	CTCATGTCAACGGAGCACAC
GRMZM2G426953_T01	DQ855284.1	rbohA	respiratory burst oxidase protein A	CAGCGCACACAAGAACTCTC	CCCCGCATACATCAAAACTT
GRMZM2G138152_T03	EU807966.1	rbohB	respiratory burst oxidase protein B	TTGGGTTACACGTGAGCAAG	AATGGAGCAAAGGCAACTGT
GRMZM2G043435_T01	DQ897930.1	rbohC	respiratory burst oxidase protein C	GGCACAGGAACTAAGCAAGC	AAACTCATCGCCAAGAAAGC
GRMZM2G032852_T03	AY109304.1	CaK	Calcium-dependent protein kinase	AACCACTTCCCAAGGAGACC	CTGTGCGTCAGGAACTTGC
GRMZM2G015933_T01	EU954960.1	BRI1	BRASSINOSTEROID INSENSITIVE 1-associated receptor kinase 1 precursor	TCCTCTCCCTTGTCGTTGTT	AGCCTTGATCCAGGACTCTTC
GRMZM2G002100_T01	EU965114.1	MAPK6	mitogen-activated protein kinase 6	CACACCCTTACTTGGCATCA	ATCACCGGCTGAAATTGAAC
GRMZM2G106928_T01	EU959272.1	SOD	superoxide dismutase	CAGCGCACACAAGAACTCTC	CCCCGCATACATCAAAACTT
GRMZM2G089850_T01	NM_001156658.1	DOF	dof zinc finger protein MNB1A	CTCCTGCTTTGCTCTGCTCT	AATGGAGCAAAGGCAACTGT
GRMZM2G400470_T03	BT065734.1	MAPKK(1/2)	Mitogen-Activated Protein Kinase Kinase (1/2)	CAACGAGCTTGTGGAGAACA	TCTGACCGTCCTGGTAGTCC
GRMZM2G384293_T02	NM_001174573	NOA1	putative nitric oxide synthase	ATTCTACCTTCCGTGCGTGA	GACAACCCAGTCGCCTATACA

Table S2
Merged effects of different chemicals interfering with NO biosynthesis/scavenging and nitrate supply/depletion on root and leaf fresh weight.

Treatment	Total Root Weight (g)	Leaf Weight (g)
**+NO_3^-**	0.07 *a*	0.131 *a*
**+ NO_3^- +cPTIO**	0.057 *bc*	0.124 *a*
**+ NO_3^- +W**	0.049 *c*	0.127 *a*
**+ NO_3^- + L-NAME**	0.069 *a*	0.131 *a*
**+ NH_4^+**	0.057 *bc*	0.128 *a*
**- NO_3^-**	0.051 *bc*	0.116 *a*
**- NO_3^- +SNP**	0.059 *b*	0.132 *a*
	$P < 0.01$	ns
